# Association Between Changes in Alcohol Consumption Before and After the Great East Japan Earthquake and Risk of Hypertension: A Study Using the Ministry of Health, Labour and Welfare National Database

**DOI:** 10.2188/jea.JE20220161

**Published:** 2023-12-05

**Authors:** Haruka Sato, Eri Eguchi, Narumi Funakubo, Hironori Nakano, Hironori Imano, Tetsuya Ohira

**Affiliations:** 1 Department of Epidemiology, Fukushima Medical University School of Medicine, Fukushima, Japan; 2 Department of Public Health, Kindai University Faculty of Medicine, Osaka, Japan; 3 Radiation Medical Science Center for the Fukushima Health Management Survey, Fukushima Medical University, Fukushima, Japan

**Keywords:** Great East Japan Earthquake, disaster, hypertension, excessive alcohol drinking, National Database

## Abstract

**Background:**

The 2011 Great East Japan Earthquake has resulted in a nuclear accident, forcing residents of the surrounding areas to evacuate. To determine any association between excessive drinking and hypertension in the setting of disaster, we assessed whether the proportion of excessive drinkers increased and if post-disaster excessive drinking was a risk factor for hypertension.

**Methods:**

This retrospective study assessed data from the Japanese National Database. Cumulative population data for Fukushima Prefecture (3,497,576 people) were analyzed by categorizing residents into four areas—evacuation, coastal, central, and mountainous—to calculate the proportion of excessive, heavy (equivalent to binge drinking), and at-risk drinkers for 2008–2017. The hazard ratios (HRs) for the incidence of hypertension for 2012–2017 were examined in association with changes in drinking status pre- and post-disaster, which included 136,404 people who received specific health checkups pre-disaster (2008–2010) and post-disaster (2011–2012).

**Results:**

The proportion of excessive drinkers among women increased after the disaster in all areas examined. The association between excessive drinking and the incidence of hypertension was determined among men and women in all areas; it was stronger among women in the evacuation areas, with the sex- and age-adjusted HRs for the incidence of hypertension of 1.41 for pre-disaster excessive drinking, 2.34 for post-disaster excessive drinking, and 3.98 for pre- and post-disaster excessive drinking, compared with not excessive drinking pre- and post-disaster.

**Conclusion:**

Excessive drinking post-disaster may be associated with an increased risk of hypertension among men and women, especially among women in the evacuation areas.

## INTRODUCTION

The Great East Japan Earthquake was a compound disaster of an earthquake, a tsunami, and a nuclear power plant accident. The tsunami struck the Fukushima Daiichi Nuclear Power Plant (NPP), which resulted in the subsequent release of radiation, and more than 160,000 residents who lived in the areas surrounding the NPP had to evacuate from their homes because of radiation hazards.

Natural disasters are known to be a major source of stress^1^^–^^3^ and are associated with alcohol abuse,^4^^,^^5^ increased prevalence of mental disorders,^6^ and higher suicidal ideation.^7^ Typically, the experience of stressful life events is linked to increased consumption of alcohol.^8^ However, not all studies have reported that natural disasters were associated with excessive drinking—whereas some studies showed an association between exposure to traumatic events and increased alcohol consumption^9^^,^^10^—several other studies did not show any association.^11^^,^^12^

According to the Fukushima Health Management Survey, which was created to examine the chronic impact of disaster,^13^ the prevalence of lifestyle diseases^14^^–^^17^ increased in the evacuation areas compared with some non-evacuation areas. The survey also reported that, among residents of evacuation areas, trauma reactions were risk factors for excessive drinking.^18^ In addition, psychological distress and trauma reactions were found to increase the risk of new cases of stroke and heart disease after the disaster.^19^

It has been well-reported that excessive alcohol drinking causes an increase in blood pressure,^20^^,^^21^ and hypertension is significantly associated with cardiovascular disease (CVD).^22^^,^^23^ Based on these findings, we hypothesized that after the disaster, the proportion of excessive drinkers would increase, especially in the evacuation areas, and that excessive drinking would be a risk for an increased incidence of hypertension. However, the association of change in drinking status before and after the disaster with incidence of hypertension has not been examined. Therefore, in this study, we examined the trends in the proportion of excessive drinkers from 2008 to 2017 and the association of change in excessive drinking status before and after the disaster with incidence of hypertension in evacuees and non-evacuees in Fukushima Prefecture.

## METHODS

### Study participants

This study used data from the National Database (NDB) of the Japanese Ministry of Health, Labour and Welfare, which collects information on specific health checkups for all medical insurance subscribers between ages 40 and 74 years based on the Securing Medical Care for the Elderly Act. These health checkups are intended to identify persons who require specific health guidance to decrease the rate of or risk for metabolic syndrome.^24^

**Figure 1.**
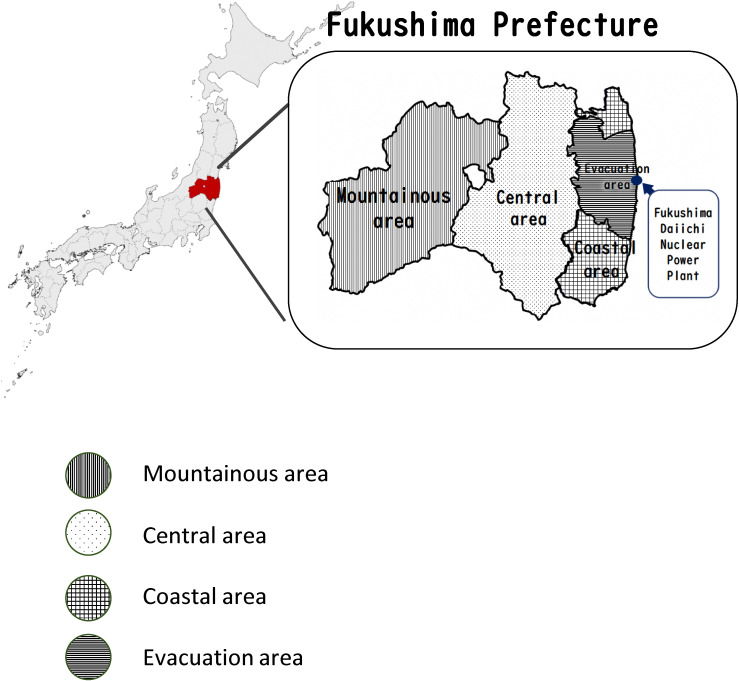
Geographic details of the mountainous area, the central area, the coastal area, the evacuation area, and the Fukushima Daiichi Nuclear Power Plant

The target population was all residents living in Fukushima Prefecture who received an annual specific health checkup between April 1, 2008, and March 31, 2017. Specific health checkups are performed on a fiscal year; checkups from April 1, 2008 to March 31, 2009 are included in 2008. Fukushima Prefecture was divided into four areas: evacuation, coastal, central, and mountainous areas (Figure 1). There were 12 evacuation areas (the cities of Tamura and Minamisōma; the towns of Kawamata, Hirono, Naraha, Tomioka, Okuma, Futaba, and Namie; and the villages of Kawauchi, Katsurao, and Iitate); these areas were designated by the government as an evacuation area after the NPP accident because of the expected high level radiation exposure (>20 msv/year). In the coastal area, many people reportedly died due to the tsunami, and most of the municipalities in the evacuation areas were located here before the disaster. In the central area, this was where the earthquake was heavily felt, causing landslides, dam breaches, and building collapses; meanwhile, the mountainous area was far from the epicenter of the earthquake; thus, the direct impact was limited.

The following are the documented annual populations of Fukushima Prefecture, as residents are listed to undergo health checkups each fiscal year, from 2008 to 2017: 933,638 (2008); 929,111 (2009); 923,732 (2010); 914,725 (2011); 910,714 (2012); 909,545 (2013); 912,479 (2014); 911,308 (2015); 908,719 (2016); and 903,532 (2017). In total, 3,866,674 study participants have undergone health checkup during this 10-year period.

First, to identify the trends in the proportion of excessive drinkers by four geographic areas, we excluded 369,098 participants because of missing data for the amount and frequency of alcohol consumption. Thus, the final number of study participants from 2008 to 2017 was 3,497,576.

**Figure 2.**
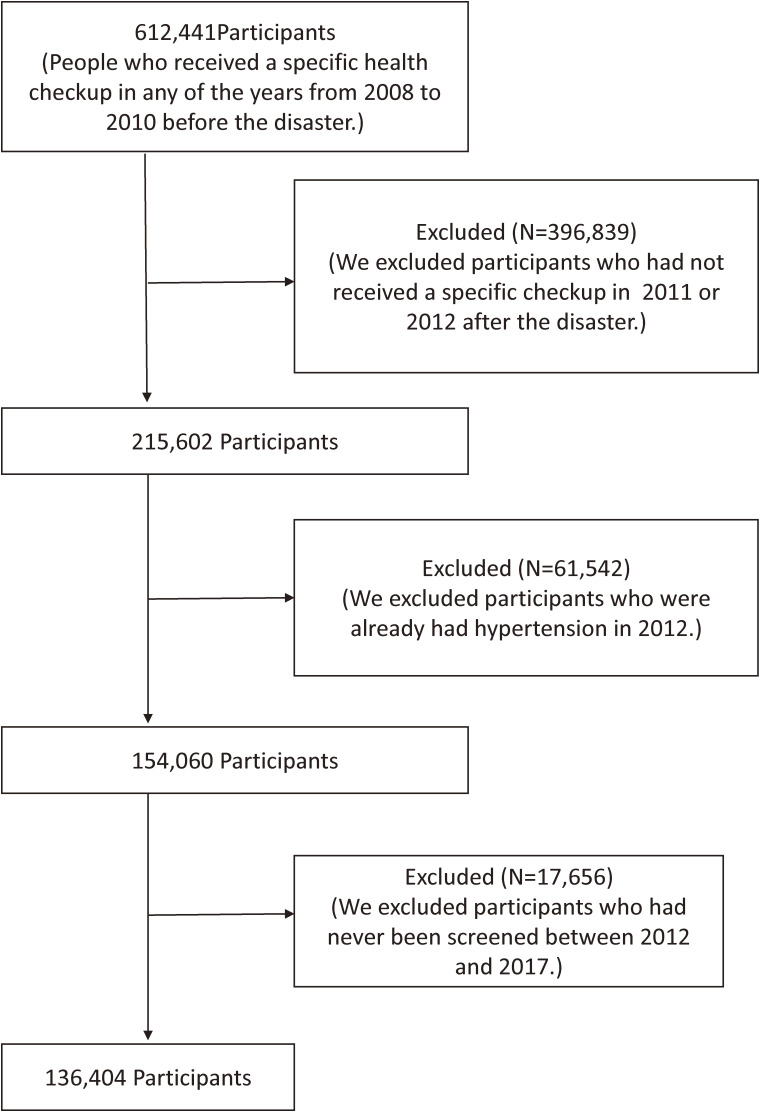
Selection of study participants to to examine the change in terms of excessive drinking status before and after the disaster with the incidence of hypertension after the disaster Participants were selected to use Cox proportional hazards models to calculate sex- and age-adjusted hazard ratios (HRs) and multivariable-adjusted HRs and 95% confidence intervals (CIs) for the incidence of post-disaster hypertension associated with changes in excessive drinking status. The HRs of the groups who drank excessively before (2008–2010), after (2011–2012), and before and after the disaster were calculated, with reference to the groups who did not drink excessively before and after the disaster.

Second, examining the change in terms of excessive drinking status before and after the disaster with the incidence of hypertension after the disaster longitudinally, we targeted residents who underwent a specific health checkup before (in any of the years from 2008 to 2010) and after (in any of the years from 2011 to 2012) the disaster (baseline), and at least one checkup between 2012 and 2017 (follow-up). We used ID1N, an NDB individual identification variable, to track the participants. ID1N is an ID generated to link receipt information with specific health checkup and specific health guidance information, based on the Insurer’s number, symbol, number of insured certificate, date of birth, and sex. In the follow-up period, 61,542 participants who had already developed hypertension in 2012 were excluded (Figure 2). For pre-disaster (2008–2010) data on drinking status, 2010 data were used first, and 2009 data were used when 2010 data were not available; moreover, data from 2008 were used when data from 2010 and 2009 were unavailable. For post-disaster (2011–2012) data on drinking status, 2011 data were used first, and 2012 data were used when 2011 data were not available.

We applied to the Ministry of Health, Labour and Welfare for use of the NDB and were granted permission. The study protocol and waiver of informed consent were approved by the Ethics Committee of the Fukushima Medical University (#30225), in accordance with ethical guidance for medical research involving human subjects.

### Measures and definitions

A self-administered “standard questionnaire”^25^ was used for other lifestyle assessments. Data on the frequency and amount of alcohol consumption were stored as categorical variables in the NDB. Regarding the frequency of alcohol consumption, participants select one of the following three items: (1) never (cannot drink), (2) occasionally, and (3) every day. Regarding the amount of alcohol consumed per day, they select one of the following three items that were expressed using the traditional Japanese unit of volume: (1) <1 “go,” (2) 1–2 “go,” (3) 2–3 “go,” and (4) ≥3 “go,”. One “go” contains 22 g of ethanol, corresponding to approximately “2 drinks,” and is equivalent to 180 mL of sake, 1 bottle (500 mL) of beer, 2 single shots (60 mL) of whiskey, or 2 glasses (240 mL) of wine. To be consistent with previous research,^26^^,^^27^ participants who drank every day and >2 “go” (≥44 g of ethanol) of alcohol, which are equivalent to “4 drinks,” were classified as excessive drinkers. Heavy drinkers in men was defined as drinking 3 “go” (≥66 g of ethanol), which is equivalent to “6 drinks.” Heavy drinking approximately corresponds to binge drinking^28^ or heavy episodic drinking.^29^ At-risk drinkers in women was defined as drinking 1 “go” (≥22 g of ethanol), which is equivalent to “2 drinks.” Based on their drinking status, the participants were divided into four groups: (1) not excessive drinking pre-disaster (2008–2010) and post-disaster (2011–2012); (2) excessive drinking pre-disaster, but not post-disaster; (3) excessive drinking post-disaster, but not pre-disaster; and (4) excessive drinking pre- and post-disaster.

Systolic and diastolic blood pressure data were stored as continuous variables, and the data on the use of anti-hypertensive medication were stored as categorical variables in the NDB. Hypertension was defined as a systolic blood pressure of at least 140 mm Hg, diastolic blood pressure of at least 90 mm Hg, or the use of anti-hypertensive medication.^30^ Blood pressure was measured after resting while sitting for at least 5 minutes, avoiding conditions that affect blood pressure measurement such as exercise and conversation based on a standardized protocol for the specific health checkup.^25^ The measurement was performed at least twice with an interval of at least 1 minute, and the mean value was used. The use of anti-hypertensive medication was examined using a questionnaire, and the validation of self-reported anti-hypertensive medication in the health checkup was high.^31^

A standard questionnaire was used for other lifestyle assessments, which included information on “current smoking” (whether or not a person habitually smokes), “regular exercise” (whether or not a person continues light, sweaty exercise for at least 30 minutes, 2 days a week, for at least 1 year), and “sleeping habits” (whether or not a person gets enough rest from sleep). Height in stocking feet and weight in light clothing were measured, and body mass index (BMI) was calculated as weight (kg)/height (m)^2^.

### Statistical analysis

We calculated the sex- and age-adjusted proportion of excessive drinkers in Fukushima Prefecture by geographic area for men and women during 2008–2017. Seven 5-year age groups (40–44, 45–49, 50–54, 55–59, 60–64, 65–69, and 70–74 years) were used for the age adjustment. The prevalence ratios of excessive drinking in the post-disaster period (2011–2017), compared with the pre-disaster period (2008–2010), were calculated by geographic area using sex- and age-adjusted Poisson regression analysis.

Cox proportional hazards models were used to calculate sex- and age-adjusted hazard ratios (HRs) and multivariable HRs and 95% confidence intervals (CIs) for the incidence of post-disaster hypertension associated with changes in excessive drinking status. The HRs of the groups who drank excessively before (2008–2010), after (2011–2012), and before and after the disaster were calculated, with reference to the groups who did not drink excessively before and after the disaster. The multivariable-adjusted covariates in the Cox proportional hazards models were BMI (continuous), current smoking (yes/no), regular exercise (yes/no), and sleeping habits (yes/no). Data from 2011 were first used, and if deemed unavailable, 2012 data were used.

All analyses were conducted by sex and age categories (≥60 years and <60 years). The proportion and HRs of heavy drinking (≥66 g of ethanol) in men and at-risk drinking (≥22 g of ethanol) in women were analyzed through a sensitivity analysis. The software package SAS version 9.4 (SAS Institute, Cary, NC, USA) was used for analyses. All probability values for statistical tests were two-tailed, and *P*-values <0.05 were considered statistically significant.

## RESULTS

Characteristics of participants who received the specific health checkup annually from 2008 to 2017 are shown in Table 1. The average proportion of men was 51.8%. The proportion of people older than 60 years was the lowest in the central area, whereas it was the highest in the evacuation areas. Moreover, the participation rate for health checkups increased yearly from 36.1% in 2008 to 48.7% in 2017.

**Table 1.** The characteristics of participants who underwent the specific health checkup from 2008 to 2017

	2008	2009	2010	2011	2012	2013	2014	2015	2016	2017
Total										
Numbers										
Mountainous area	43,459	50,214	52,834	54,428	56,428	56,720	59,576	61,278	61,946	62,457
Central area	156,183	185,956	188,187	195,704	201,938	206,827	217,322	225,065	231,462	238,389
Coastal area	42,269	53,141	54,898	52,313	58,249	52,743	55,922	68,439	71,144	73,538
Evacuation areas	29,545	34,919	34,146	24,231	28,236	28,954	30,301	32,015	32,481	33,719
Proportion of participants^a^, %										
Mountainous area	30.9	36.2	38.6	40.2	42.2	42.8	45.0	46.6	47.6	48.3
Central area	30.5	36.3	36.8	38.3	39.6	40.4	42.2	43.4	44.5	45.8
Coastal area	23.1	29.1	30.2	29.0	32.6	29.4	31.1	38.0	39.5	41.0
Evacuation areas	30.2	36.1	35.6	25.5	30.5	31.5	33.1	34.9	35.6	37.3
Men^b^, %										
Mountainous area	46.9	47.9	48.1	49.0	49.4	49.5	50.1	50.6	50.6	50.8
Central area	52.2	52.3	52.1	52.3	52.5	52.6	52.9	52.6	52.7	52.5
Coastal area	50.4	52.1	52.4	53.0	53.3	52.6	53.2	53.7	53.7	53.8
Evacuation areas	47.0	48.9	48.7	51.4	50.9	50.9	50.4	50.4	50.8	50.9
Age ≥60 years^c^, %										
Mountainous area	47.4	43.7	43.1	43.6	43.4	44.3	43.8	43.6	43.7	44.1
Central area	39.4	36.9	37.8	37.9	38.6	39.1	39.3	39.6	39.5	39.5
Coastal area	42.3	37.6	39.0	38.0	37.3	41.0	41.7	39.0	38.6	38.6
Evacuation areas	46.8	43.1	44.1	42.7	47.6	49.4	50.2	49.8	50.6	51.6
Men										
Numbers										
Mountainous area	20,166	24,030	25,437	26,682	27,930	28,089	29,863	31,079	31,394	31,770
Central area	81,305	97,436	98,118	102,503	106,127	108,779	114,860	118,271	121,800	124,982
Coastal area	21,085	27,749	28,762	27,809	31,241	27,662	29,612	36,793	38,308	39,658
Evacuation areas	13,868	17,157	16,674	12,525	14,365	14,711	15,230	16,114	16,474	17,114
Age ≥60 years^c^, %										
Mountainous area	42.5	39.8	39.0	39.4	39.5	40.6	40.3	40.2	40.7	41.4
Central area	33.5	32.3	33.6	33.8	34.7	35.1	35.5	35.9	36.1	36.2
Coastal area	36.3	32.0	33.4	32.9	32.5	35.4	36.5	34.7	34.7	35.0
Evacuation areas	44.4	40.9	42.1	40.0	44.8	45.9	47.2	47.5	47.7	48.7
Women										
Numbers										
Mountainous area	23,293	26,184	27,397	27,746	28,498	28,631	29,713	30,199	30,552	30,687
Central area	74,878	88,520	90,069	93,201	95,811	98,048	102,462	106,794	109,662	113,407
Coastal area	21,184	25,392	26,136	24,504	27,008	25,081	26,310	31,646	32,836	33,880
Evacuation areas	15,677	17,762	17,472	11,706	13,871	14,243	15,071	15,901	16,007	16,605
Age ≥60 years^c^, %										
Mountainous area	52.1	47.6	47.0	47.6	47.2	47.9	47.2	46.9	46.6	46.8
Central area	46.0	42.0	42.4	42.5	42.9	43.5	43.5	43.6	43.3	43.1
Coastal area	48.9	43.9	45.3	43.7	42.8	47.2	47.6	43.8	42.9	42.6
Evacuation areas	49.1	45.3	46.1	45.4	50.4	52.9	53.2	52.0	53.5	54.5

^a^The percentages were adjusted for sex and age.

^b^The percentages were adjusted for age.

^c^The percentages were adjusted for sex.

**Figure 3.**
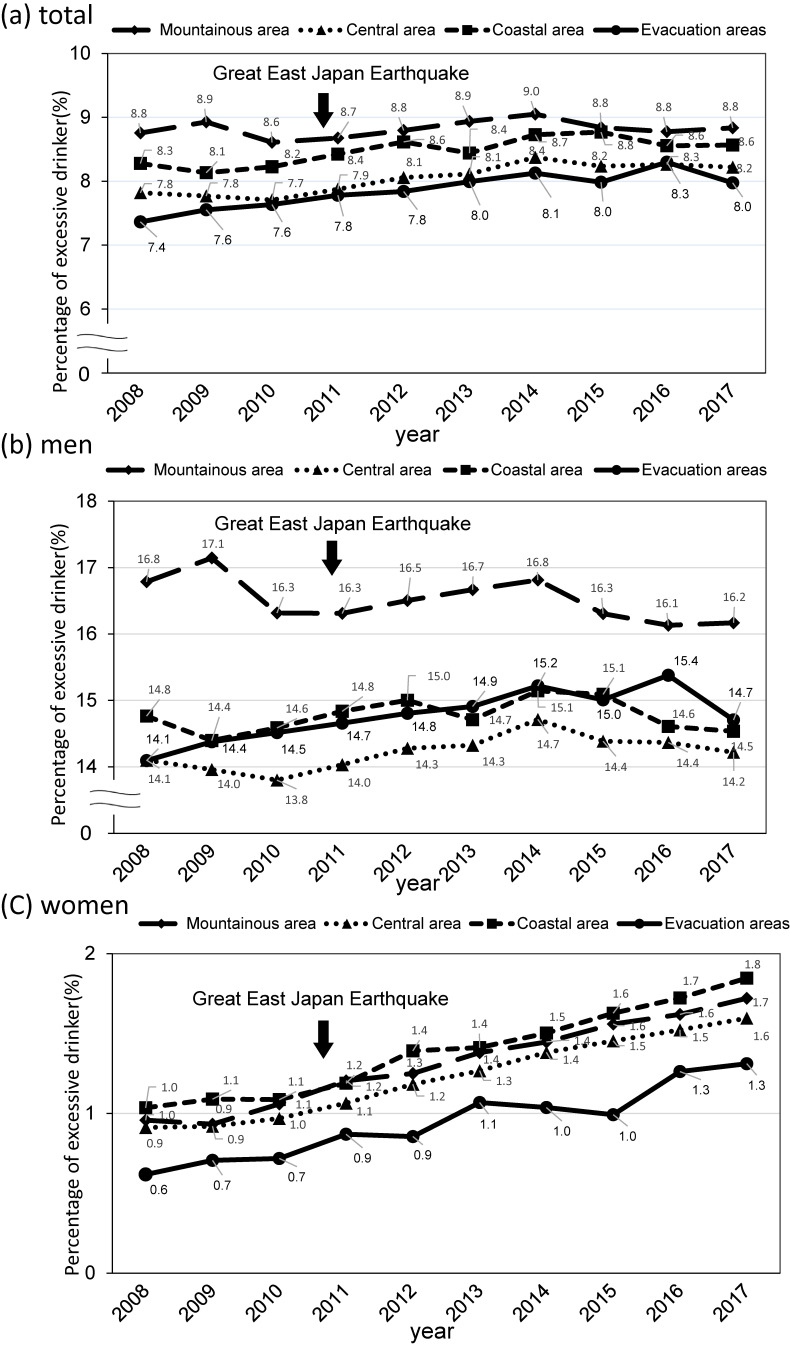
Age-sex adjusted proportion of excessive drinkers by geographic area from 2008 to 2017 (a) total, (b) men, and (c) women of the sex- and age-adjusted proportion of excessive drinkers in Fukushima Prefecture by geographic area for men and women during 2008–2017. Participants who drank more than 2 “go” (44 g or more of ethanol) of alcohol every day were classified as excessive drinkers

Figure 3 shows the proportion of excessive drinkers by geographic area from 2008 to 2017. For men, the rate was almost unchanged before and after the disaster in all areas. For women in the evacuation areas, the rate was 0.7% in 2010 before the disaster, but it increased to 0.9% in 2012 and 1.3% in 2017 after the disaster. Among women, there were similar upward trends in other areas as well. The proportion of heavy drinkers (≥66 g of ethanol) in men by area from 2008 to 2017 is shown in eFigure 1. In the evacuation areas, the rate decreased once in 2011 after the disaster, but it increased thereafter. The proportion of at-risk drinkers (≥22 g of ethanol) in women by area from 2008 to 2017 is shown in eFigure 2. All areas showed an increasing trend.

Table 2 shows the prevalence ratios of excessive drinkers after the disaster compared with before the disaster (2008–2010). The prevalence ratios in 2012 in men and group younger than 60 years were relatively the same as those before the disaster in all areas. The prevalence ratios of women in 2012 were significantly higher at 1.25 (95% CI, 1.02–1.54) in the evacuation areas, 1.32 (95% CI, 1.17–1.49) in the coastal area, 1.28 (95% CI, 1.19–1.37) in the central area, and 1.30 (95% CI, 1.15–1.48) in the mountainous area. In the group older than 60 years, there were similar upward trends as seen in women in all areas as well.

**Table 2.** The prevalence ratios (95% CIs) of excessive drinkers^a^ after the Great East Japan Earthquake compared to that before the disaster (2008–2010)

	2008–2010	2011	2012	2013	2014	2015	2016	2017
Total								
Mountainous area	1.00	1.00 (0.96–1.03)	1.01 (0.98–1.05)	1.03 (0.99–1.06)	1.04 (1.00–1.07)	1.01 (0.98–1.05)	1.00 (0.97–1.04)	1.01 (0.98–1.04)
Central area	1.00	1.02 (1.00–1.04)	1.04 (1.02–1.06)	1.05 (1.03–1.06)	1.08 (1.06–1.10)	1.06 (1.04–1.08)	1.06 (1.04–1.08)	1.05 (1.04–1.07)
Coastal area	1.00	1.03 (1.00–1.07)	1.06 (1.02–1.09)	1.02 (0.99–1.06)	1.06 (1.02–1.09)	1.07 (1.03–1.10)	1.04 (1.01–1.07)	1.04 (1.01–1.07)
Evacuation areas	1.00	1.04 (0.99–1.09)	1.05 (1.00–1.10)	1.06 (1.01–1.11)	1.07 (1.03–1.12)	1.06 (1.01–1.11)	1.09 (1.05–1.14)	1.05 (1.00–1.09)
Men								
Mountainous area	1.00	0.98 (0.95–1.02)	0.99 (0.96–1.03)	1.00 (0.97–1.03)	1.01 (0.97–1.04)	0.98 (0.94–1.01)	0.96 (0.93–1.00)	0.96 (0.93–0.99)
Central area	1.00	1.01 (0.99–1.03)	1.03 (1.01–1.05)	1.03 (1.01–1.04)	1.05 (1.03–1.07)	1.02 (1.01–1.04)	1.02 (1.01–1.04)	1.01 (0.99–1.03)
Coastal area	1.00	1.03 (0.99–1.06)	1.04 (1.00–1.07)	1.00 (0.97–1.04)	1.03 (1.00–1.07)	1.03 (1.00–1.07)	1.00 (0.97–1.03)	0.99 (0.96–1.03)
Evacuation areas	1.00	1.03 (0.98–1.08)	1.04 (0.99–1.09)	1.04 (0.99–1.09)	1.05 (1.01–1.11)	1.04 (0.99–1.09)	1.06 (1.01–1.11)	1.01 (0.96–1.06)
Women								
Mountainous area	1.00	1.25 (1.10–1.43)	1.30 (1.15–1.48)	1.43 (1.27–1.62)	1.50 (1.33–1.69)	1.61 (1.43–1.80)	1.65 (1.48–1.85)	1.75 (1.57–1.96)
Central area	1.00	1.15 (1.07–1.24)	1.28 (1.19–1.37)	1.37 (1.28–1.46)	1.49 (1.39–1.59)	1.56 (1.46–1.66)	1.62 (1.52–1.73)	1.70 (1.60–1.80)
Coastal area	1.00	1.13 (0.99–1.30)	1.32 (1.17–1.49)	1.33 (1.17–1.51)	1.41 (1.25–1.59)	1.53 (1.36–1.71)	1.60 (1.44–1.79)	1.71 (1.54–1.91)
Evacuation areas	1.00	1.28 (1.03–1.58)	1.25 (1.02–1.54)	1.55 (1.28–1.88)	1.50 (1.24–1.81)	1.43 (1.19–1.73)	1.82 (1.53–2.17)	1.89 (1.60–2.24)
Age <60 years								
Mountainous area	1.00	0.97 (0.93–1.01)	0.97 (0.93–1.01)	0.98 (0.94–1.02)	0.97 (0.93–1.01)	0.95 (0.91–0.99)	0.93 (0.90–0.97)	0.94 (0.90–0.97)
Central area	1.00	1.00 (0.98–1.02)	1.03 (1.00–1.05)	1.03 (1.01–1.05)	1.04 (1.02–1.06)	1.01 (0.99–1.04)	1.01 (0.99–1.04)	0.99 (0.97–1.01)
Coastal area	1.00	0.99 (0.95–1.03)	1.01 (0.97–1.05)	0.97 (0.93–1.01)	1.00 (0.96–1.04)	1.00 (0.96–1.04)	0.98 (0.94–1.01)	0.97 (0.94–1.01)
Evacuation areas	1.00	1.04 (0.98–1.10)	1.03 (0.97–1.09)	1.03 (0.97–1.10)	1.03 (0.97–1.10)	0.99 (0.93–1.05)	1.06 (1.00–1.12)	0.98 (0.92–1.04)
Age ≥60 years								
Mountainous area	1.00	1.04 (0.98–1.11)	1.10 (1.04–1.16)	1.11 (1.05–1.18)	1.17 (1.11–1.24)	1.14 (1.08–1.20)	1.14 (1.08–1.20)	1.16 (1.10–1.22)
Central area	1.00	1.07 (1.03–1.11)	1.09 (1.05–1.12)	1.10 (1.07–1.14)	1.18 (1.14–1.21)	1.17 (1.14–1.21)	1.18 (1.14–1.22)	1.22 (1.19–1.26)
Coastal area	1.00	1.13 (1.06–1.21)	1.16 (1.09–1.24)	1.18 (1.11–1.26)	1.23 (1.16–1.30)	1.24 (1.17–1.31)	1.20 (1.14–1.27)	1.22 (1.16–1.29)
Evacuation areas	1.00	1.03 (0.95–1.13)	1.09 (1.01–1.18)	1.13 (1.05–1.22)	1.17 (1.09–1.26)	1.18 (1.10–1.27)	1.18 (1.10–1.27)	1.18 (1.11–1.27)

CI, confidence interval.

Notes: values in parentheses.

^a^Excessive drinkers was defined as drinking ≥44 g of ethanol every day.

Two “go” is equivalent to 44 g of ethanol and approximately “4 drinks.”

The sex- and age-adjusted HRs and multivariable HRs of the incidence of hypertension by area with changes in excessive drinking status pre- and post-disaster are shown in Table 3. For men in the evacuation areas, the sex- and age-adjusted HRs, compared with those who did not drink excessively pre- and post-disaster (81.3%), were 1.40 (95% CI, 1.19–1.64) for those who drank excessively pre-disaster but not post-disaster (4.3%), 1.45 (95% CI, 1.25–1.69) for those who drank excessively post-disaster but not pre-disaster (5.2%), and 1.53 (95% CI, 1.37–1.71) for those who drank excessively pre- and post-disaster (9.2%). Among men and the group younger than 60 years, HRs were significantly higher in the group with excessive drinking, regardless of changes in excessive drinking status. For women in the evacuation areas, the sex- and age-adjusted HRs, compared with those who did not drink excessively pre- and post-disaster (98.2%), were 1.41 (95% CI, 0.35–5.69) for those who drank excessively pre-disaster but not post-disaster (0.4%), 2.34 (95% CI, 1.04–5.24) for those who drank excessively post-disaster but not pre-disaster (0.8%), and 3.98 (95% CI, 1.77–8.94) for those who drank excessively pre- and post-disaster (0.7%). Among women and the group older than 60 years from the central and coastal areas, the sex- and age-adjusted HRs were determined to be significantly higher in the group with excessive drinking status pre- and post-disaster. After adjusting for confounding variables, the association of HRs was found to be mostly similar to the sex- and age-adjusted HRs. The age-adjusted HRs and multivariable HRs of the incidence of hypertension by area of heavy drinkers (≥66 g of ethanol) in men are shown in eTable 1. Among men, the HRs were significantly higher in the group with heavy drinking status post-disaster, and pre- and post-disaster. The age-adjusted HRs and multivariable HRs of the incidence of hypertension by area of at-risk drinkers (≥22 g of ethanol) in women are shown in eTable 2. In women, the HRs were significantly higher in the group with an at-risk drinking status post-disaster, and pre- and post-disaster.

**Table 3.** HRs (95% CIs) of the incidence of hypertension by areas with changes in excessive drinking^a^ status pre- and post-disaster

	Neither^b^	Pre-disaster^c^	Post-disaster^d^	Both^e^
Total				
Mountainous area (*n*)	15,170 (89.0%)	388 (2.3%)	479 (2.8%)	1,012 (5.9%)
Sex- and age-adjusted HR^f^	1.00	1.28 (1.02–1.59)	1.47 (1.21–1.78)	1.76 (1.55–2.01)
Multivariable-adjusted HR^g^	1.00	1.31 (1.04–1.65)	1.61 (1.32–1.97)	1.91 (1.67–2.19)
Central area (*n*)	74,978 (89.8%)	1,960 (2.6%)	2,219 (2.7%)	4,371 (5.2%)
Sex- and age-adjusted HR	1.00	1.52 (1.38–1.67)	1.55 (1.42–1.69)	1.55 (1.46–1.66)
Multivariable-adjusted HR	1.00	1.51 (1.37–1.67)	1.64 (1.49–1.79)	1.69 (1.58–1.80)
Coastal area (*n*)	25,081 (90.1%)	629 (2.3%)	794 (2.9%)	1,339 (4.8%)
Sex- and age-adjusted HR	1.00	1.39 (1.19–1.62)	1.47 (1.28–1.70)	1.56 (1.40–1.74)
Multivariable-adjusted HR	1.00	1.42 (1.20–1.67)	1.49 (1.27–1.74)	1.64 (1.47–1.83)
Evacuation areas (*n*)	7,074 (88.6%)	207 (2.6%)	262 (3.3%)	441 (5.5%)
Sex- and age-adjusted HR	1.00	1.41 (1.03–1.93)	1.60 (1.21–2.11)	1.88 (1.54–2.29)
Multivariable-adjusted HR	1.00	1.53 (1.10–2.13)	1.68 (1.26–2.23)	2.04 (1.66–2.50)
Men				
Mountainous area (*n*)	6,877 (80.3%)	336 (3.9%)	422 (4.9%)	925 (10.8%)
Sex- and age-adjusted HR	1.00	1.26 (1.00–1.59)	1.46 (1.19–1.79)	1.70 (1.48–1.95)
Multivariable-adjusted HR	1.00	1.30 (1.02–1.66)	1.60 (1.30–1.98)	1.84 (1.60–2.13)
Central area (*n*)	36,583 (82.5%)	1,759 (4.0%)	1,936 (4.4%)	4,093 (9.2%)
Sex- and age-adjusted HR	1.00	1.52 (1.37–1.68)	1.54 (1.41–1.69)	1.55 (1.45–1.65)
Multivariable-adjusted HR	1.00	1.51 (1.36–1.68)	1.62 (1.47–1.78)	1.68 (1.57–1.80)
Coastal area (*n*)	12,207 (82.9%)	558 (3.8%)	705 (4.8%)	1,247 (8.5%)
Sex- and age-adjusted HR	1.00	1.40 (1.19–1.64)	1.45 (1.25–1.69)	1.53 (1.37–1.71)
Multivariable-adjusted HR	1.00	1.45 (1.22–1.72)	1.47 (1.25–1.73)	1.61 (1.43–1.80)
Evacuation areas (*n*)	3,670 (81.3%)	194 (4.3%)	236 (5.2%)	416 (9.2%)
Sex- and age-adjusted HR	1.00	1.40 (1.19–1.64)	1.45 (1.25–1.69)	1.53 (1.37–1.71)
Multivariable-adjusted HR	1.00	1.56 (1.11–2.19)	1.58 (1.17–2.14)	1.94 (1.57–2.39)
Women				
Mountainous area (*n*)	8,293 (97.7%)	52 (0.6%)	57 (0.7%)	87 (1.0%)
Sex- and age-adjusted HR	1.00	1.33 (0.67–2.68)	1.46 (0.73–2.92)	2.55 (1.70–3.82)
Multivariable-adjusted HR	1.00	1.33 (0.66–2.67)	1.54 (0.76–3.10)	2.61 (1.71–3.97)
Central area (*n*)	38,395 (98.1%)	201 (0.5%)	283 (0.7%)	278 (0.7%)
Sex- and age-adjusted HR	1.00	1.54 (1.09–2.18)	1.57 (1.16–2.13)	1.66 (1.23–2.24)
Multivariable-adjusted HR	1.00	1.48 (1.01–2.19)	1.77 (1.30–2.41)	1.82 (1.33–2.48)
Coastal area (*n*)	12,874 (98.1%)	71 (0.5%)	89 (0.7%)	92 (0.7%)
Sex- and age-adjusted HR	1.00	1.59 (0.96–2.64)	1.56 (0.95–2.55)	2.05 (1.37–3.07)
Multivariable-adjusted HR	1.00	1.50 (0.87–2.60)	1.56 (0.92–2.66)	2.18 (1.44–3.30)
Evacuation areas (*n*)	3,404 (98.2%)	13 (0.4%)	26 (0.8%)	25 (0.7%)
Sex- and age-adjusted HR	1.00	1.41 (0.35–5.69)	2.34 (1.04–5.24)	3.98 (1.77–8.94)
Multivariable-adjusted HR	1.00	1.33 (0.32–5.45)	2.22 (0.97–5.07)	4.27 (1.88–9.69)
Age <60 years				
Mountainous area (*n*)	13,571 (88.8%)	350 (2.3%)	441 (2.9%)	915 (6.0%)
Sex- and age-adjusted HR	1.00	1.31 (1.05–1.64)	1.48 (1.21–1.81)	1.78 (1.55–2.03)
Multivariable-adjusted HR	1.00	1.34 (1.07–1.69)	1.66 (1.35–2.04)	1.95 (1.70–2.24)
Central area (*n*)	61,263 (89.3%)	1,643 (2.4%)	1,950 (2.8%)	3,765 (5.5%)
Sex- and age-adjusted HR	1.00	1.53 (1.38–1.69)	1.57 (1.44–1.72)	1.55 (1.45–1.66)
Multivariable-adjusted HR	1.00	1.52 (1.37–1.69)	1.67 (1.52–1.83)	1.69 (1.58–1.81)
Coastal area (*n*)	17,780 (89.1%)	444 (2.2%)	655 (3.3%)	1,079 (5.4%)
Sex- and age-adjusted HR	1.00	1.51 (1.26–1.81)	1.51 (1.29–1.77)	1.61 (1.43–1.81)
Multivariable-adjusted HR	1.00	1.70 (1.39–2.07)	1.58 (1.33–1.88)	1.75 (1.54–1.98)
Evacuation areas (*n*)	6,295 (88.7%)	176 (2.5%)	229 (3.2%)	396 (5.6%)
Sex- and age-adjusted HR	1.00	1.45 (1.04–2.01)	1.57 (1.18–2.10)	1.86 (1.52–2.27)
Multivariable-adjusted HR	1.00	1.64 (1.16–2.31)	1.65 (1.23–2.23)	2.02 (1.64–2.50)
Age ≥60 years				
Mountainous area (*n*)	1,599 (90.2%)	38 (2.1%)	38 (2.1%)	97 (5.8%)
Sex- and age-adjusted HR	1.00	0.41 (0.06–2.94)	1.27 (0.55–2.92)	1.56 (0.89–2.72)
Multivariable-adjusted HR	1.00	0.38 (0.05–2.72)	1.05 (0.42–2.61)	1.52 (0.85–2.74)
Central area (*n*)	13,715 (92.0%)	317 (2.1%)	269 (1.8%)	606 (4.1%)
Sex- and age-adjusted HR	1.00	1.37 (0.93–2.03)	1.26 (0.88–1.79)	1.62 (1.26–2.07)
Multivariable-adjusted HR	1.00	1.35 (0.89–2.06)	1.31 (0.91–1.88)	1.63 (1.27–2.10)
Coastal area (*n*)	7,301 (92.6%)	185 (2.4%)	139 (1.8%)	260 (3.3%)
Sex- and age-adjusted HR	1.00	1.22 (0.92–1.62)	1.31 (0.94–1.82)	1.37 (1.08–1.73)
Multivariable-adjusted HR	1.00	1.13 (0.84–1.50)	1.27 (0.91–1.77)	1.36 (1.07–1.73)
Evacuation areas (*n*)	779 (87.7%)	31 (3.5%)	33 (3.7%)	45 (5.1%)
Sex- and age-adjusted HR	1.00	1.14 (0.35–3.72)	1.96 (0.70–5.49)	2.20 (0.67–7.18)
Multivariable-adjusted HR	1.00	1.07 (0.32–3.56)	1.94 (0.69–5.49)	2.10 (0.63–6.97)

CI, confidence interval; HR, hazards ratio.

Notes: values in parentheses.

The hazard ratios of the incidence of hypertension with changes in excessive drinking habits were obtained by dividing the participants into four groups.

^a^Excessive drinkers was defined as drinking ≥44 g of ethanol every day.

Two “go” is equivalent to 44 g of ethanol and approximately “4 drinks.”

^b^Neither were those who were not excessive drinking pre-disaster (2008–2010) and post-disaster (2011–2012) and used as reference.

^c^Pre-disaster was defined as those who were excessive drinking pre-disaster, but not post-disaster.

^d^Post-disaster was defined as those who were excessive drinking post-disaster, but not pre-disaster.

^e^Both was defined as those who were excessive drinking pre- and post-disaster.

^f^Sex- and age-adjusted HR was adjusted for age and sex.

^g^Multivariable-adjusted HR was adjusted for age and sex, body mass index, current smoking, regular exercise, sleeping habits.

## DISCUSSION

The main findings of this study were as follows: (1) the proportion of excessive drinkers increased in women and in the group older than 60 years in all geographic areas; and (2) not only men but also women were found to have a higher risk of hypertension in the group with excessive drinking status post-disaster, and pre- and post-disaster, compared to those without excessive drinking status pre- and post-disaster.

In this study, the proportion of excessive drinkers increased among women and in the group older than 60 years in all geographic areas; this trend was also observed in the at-risk drinking group (≥22 g of ethanol). Our data support findings of previous reports showing that the proportion of excessive drinkers increased after the disaster.^9^^,^^10^ The causes of the increase in the number of excessive drinkers and at-risk drinkers in women were unclear; however, the number of women who are at-risk drinkers in Fukushima Prefecture has risen sharply compared with the results of the National Nutrition Survey.^32^ One of the reasons could be the results of the Fukushima Health Management Survey showed that sleep disturbances, trauma reactions, and a history of past mental illness were risk factors for excessive drinking among women in evacuation areas.^18^^,^^27^ Another report revealed that psychological distress was significantly and positively correlated with radiation levels in the environment.^33^ It has been reported that women are at higher risk of using alcohol to regulate negative emotions^34^ and that the older adults are at higher risk of excessive drinkers when life stage-related psychosocial stressors emerge.^35^ It was possible that the stress associated with the disaster caused excessive drinking. As of June 2021, 10 years after the disaster, more than 34,000 people are still living in places other than where they originally lived. Moreover, not only in the evacuation areas, but throughout Fukushima Prefecture, the agriculture, forestry, fisheries, and tourism industries have experienced long-term damage to their reputation after the accident at NPP.^36^ The stress caused by the change in environment may have contributed to the increase in excessive drinkers, especially among women and older adults.

Alcohol has been shown to have a complex effect on the cardiovascular system; moreover, it can even cause hypertension.^20^ In men, the risk of incidence of hypertension is higher, especially in excessive drinkers.^37^^,^^38^ In women, the risk of the incidence of hypertension is associated with higher alcohol consumption.^38^ In this study, the risk of the incidence of hypertension was significantly higher in men and group younger than 60 years with excessive drinking and heavy drinking statuses post-disaster, and pre- and post-disaster. Moreover, the risk of the incidence of hypertension was significantly higher in women with excessive drinking status pre- and post-disaster and tended to increase in the group with excessive drinking status post-disaster; the same trend was observed in the at-risk drinkers. The association continued to be significant after adjustment for BMI and other confounding factors for the risk of incidence of hypertension. Women are at a higher risk of the incidence of hypertension even with lower alcohol consumption than men.^39^ Moreover, in this study, women with at-risk drinking status post-disaster and pre- and post-disaster were also at a higher risk of the incidence of hypertension. The consumption of a large amount of alcohol is one of the important risk factors for hypertension, and the long-term effects may increase the risk of future cardiovascular disease after a disaster.

To the best of our knowledge, no longitudinal studies to date have examined the relationship between changes in excessive drinking status pre- and post-disaster and the risk of hypertension. Because disaster evacuees tend to experience psychosocial stress, the results of this study can be adapted to future disaster evacuations that may occur globally.

The strength of this study is that we used NDB data; thus, we were able to include residents throughout Fukushima Prefecture. Because the Fukushima Health Management Survey only covered municipalities that include districts designated as evacuation areas and the survey was started after the disaster, and the data included people insured by the National Health Insurance (mainly farmers, fishermen, self-employed individuals, and retirees) and did not include those insured by the Social Insurance (mainly company employees),^13^ the target population was limited. By using NDB data in this study, we could analyze a large number of people who were insured by National Health Insurance and Social Insurance and who had received specific health checkups since 2008, the year before the disaster.

Several potential limitations of this study must also be considered. First, the response rate of the specific health checkup was around 40%, which may cause a selection bias among the examinees. However, according to the National Nutrition Survey, the people who had not have the health checkup tend to be from low-income households.^40^ Individuals with lower socioeconomic status (SES) are reported to experience disproportionately greater alcohol-related health problems from similar or lower amounts of alcohol consumption compared with individuals with higher SES,^41^ and individuals with lower SES in Japan have higher odds ratios for being excessive drinkers.^42^ Therefore, it was possible that the proportion of excessive drinkers and the HRs for the incidence of hypertension associated with changes in drinking status were underestimated, but it was less likely that they were overestimated.

Second, the post-disaster increase in the specific health checkup uptake rate may indicate a change in the population undergoing specific health checkups. With the revision of the National Health Insurance Law in 2015, the Insurer Effort Support System was established. Insurers are actively encouraging residents to undergo specific health checkups, and as a result, the specific health checkup rate has increased nationwide.^43^ We were unable to find any study on screened populations because of this active encouragement. However, as a result of the active encouragement, residents who have undergone checkups tend to have the same tendency as those who have not yet undergone a checkup, and although the effect of the risk of the incidence of hypertension caused as a result of excessive drinking may be underestimated, it is unlikely to be overestimated.

Third, in a longitudinal study, the participants were tracked using ID1N of the NDB as an individual identification variable. ID1N was changed by changing the health insurer and surname. As a result, the participants could no longer be tracked and excluded in this study (Figure 2). The excluded group may have changed their residence or workplace due to evacuation after the disaster, resulting in the change of IDN1. People who have changed their residence or workplace to evacuate after a disaster are reportedly at a high risk of engaging in excessive drinking behavior.^27^ The evacuation itself was also reported to be associated with an increased risk of the incidence of hypertension.^14^ Thus, the exclusion of those without specific health checkup records may have underestimated, but not overestimated, the risk of the incidence of hypertension brought about by excessive alcohol drinking. Furthermore, comparing the basic data, the proportions of men (52.3%), women (47.7%), and those aged ≥60 years (36.2%) excluded from the analysis and those included in the analysis (50.7% [men], 49.3% [women], and 38.2% [≥60 years]) showed no significant differences.

Fourth, in this study, the 12 municipalities were defined as evacuation areas by the government because of the expected high level of radiation exposure. However, in some municipalities, such as Tamura City and Minamisōma City, not all people in the areas were evacuated, which could have underestimated the impact of the evacuation.

Fifth, this study did not adjust for all of the changes in living conditions associated with the disaster. In the Fukushima Health Management Survey, 54% of respondents in the evacuation areas changed their jobs, whereas 21% lost their jobs.^44^^,^^45^ These changes may have influenced changes in drinking status and hypertension risk. Although we could not include all the changes in the living environment in this study, we calculated the HRs of the incidence of post-disaster hypertension associated with changes in excessive drinking status, using post-disaster lifestyle habits, such as smoking, exercise, and sleeping habits, obtained from the NDB data as covariates.

Finally, we defined excessive drinking status using an annual specific health checkup result, which may lead to misclassification in some individuals. However, in previous studies, 97.5% of excessive drinkers consumed more than 44 g of ethanol daily, and 85.3% of excessive drinkers consumed the same amount of alcohol throughout the week.^46^ Therefore, the effect of single assessment was likely to be marginal.

### Conclusion

As per the findings of this study, the proportion of excessive drinkers increased in women and in the group older than 60 years in Fukushima Prefecture after the disaster. Among women, at-risk and excessive drinking statuses post-disaster may be associated with an increased risk of hypertension throughout the Fukushima Prefecture. Because excessive drinking is one of the important risk factors for hypertension, and the long-term effects may increase the risk of future cardiovascular disease after a disaster, researchers must work with local governments to take appropriate measures against excessive drinking, especially among women.

## SUPPLEMENTARY MATERIAL

eTable 1. Hazard ratios and 95% confidence intervals of the incidence of hypertension by areas with changes in heavy drinking^a^ status pre- and post-disaster in men

eTable 2. Hazard ratios and 95% confidence intervals of the incidence of hypertension by areas with changes in at-risk drinking^a^ status pre- and post-disaster in women

eFigure 1. Age- and sex-adjusted proportion of heavy drinkers (≥66 g of ethanol) in men by geographic area from 2008 to 2017

eFigure 2. Age- and sex-adjusted proportion of at-risk drinkers (≥22 g of ethanol) in women by geographic area from 2008 to 2017
