# Supplementary material for: Association Between Changes in Alcohol Consumption Before and After the Great East Japan Earthquake and Risk of Hypertension: A Study Using the Ministry of Health, Labour and Welfare National Database
Source: J Epidemiol. 2023 Dec 5;33(12):607–17. doi: 10.2188/jea.JE20220161 (PMC10635811; doi:10.2188/jea.JE20220161)
Supplement: Supplementary file 1 [file je-33-607-s001.pdf]

eTable 1. Hazard ratios and 95% confidence intervals of the incidence of hypertension by areas with changes in heavy drinking[a] status pre- and post-disaster in men

	Neither[b]	Before the disaster[c]	After the disaster[d]	Both[e]
Mountainous area (n)	18,521 (94.3%)	225 (1.6%)	621 (3.2%)	266 (1.36%)
Sex- and age-adjusted HR[f]	1.00	1.43 (1.12–1.84)	1.35 (1.17–1.57)	1.62 (1.30–2.01)
Multivariable-adjusted HR[g]	1.00	1.41 (1.10–1.82)	1.34 (1.16–1.56)	1.52 (1.21–1.90)
Central area (n)	76,646 (94.3%)	1,052 (1.3%)	2,414 (3.0%)	1,186 (1.5%)
Sex- and age-adjusted HR	1.00	1.28 (1.13–1.45)	1.33 (1.23–1.45)	1.38 (1.23–1.54)
Multivariable-adjusted HR	1.00	1.18 (1.04–1.34)	1.26 (1.16–1.37)	1.30 (1.16–1.45)
Coastal area (n)	21,462 (94.0%)	348 (1.5%)	683 (3.0%)	330 (1.5%)
Sex- and age-adjusted HR	1.00	1.45 (1.19–1.75)	1.47 (1.26–1.70)	1.53 (1.27–1.85)
Multivariable-adjusted HR	1.00	1.32 (1.07–1.63)	1.33 (1.13–1.56)	1.47 (1.20–1.79)
Evacuation areas (n)	10,389 (95.4%)	109 (1.0%)	316 (3.0%)	75 (0.7%)
Sex- and age-adjusted HR	1.00	1.39 (0.92–2.10)	1.54 (1.26–1.88)	1.52 (0.95–2.42)
Multivariable-adjusted HR	1.00	1.34 (0.87–2.06)	1.47 (1.20–1.80)	1.39 (0.87–2.22)

s

[a]Heavy drinkers in men was defined as drinking ≥66 g of ethanol per day, which is approximately corresponding to binge drinking or heavy episodic drinking. Three “go” is equivalent to 66 g of ethanol and approximately “6 drinks.”

[b]Neither were those who were not heavy drinking pre-disaster (2008–2010) and post-disaster (2011–2012) and used as reference.

[c]Pre–disaster was defined as those who were heavy drinking pre-disaster, but not post-disaster.

[d]Post–disaster was defined as those who were heavy drinking post-disaster, but not pre-disaster.

[e]Both was defined as those who were heavy drinking pre- and post-disaster.

[f]Sex- and age-adjusted HR was adjusted for age and sex.

[g]Multivariable-adjusted HR was adjusted for age and sex, body mass index, current smoking, regular exercise, sleeping habits.

eTable 2. Hazard ratios and 95% confidence intervals of the incidence of hypertension by areas with changes in at-risk drinking[a] status pre- and post-disaster in women

	Neither[b]	Before the disaster[c]	After the disaster[d]	Both[e]
Mountainous area (n)	18,796 (82.4%)	552 (2.4%)	2,438 (10.7%)	1,025 (4.5%)
Sex- and age-adjusted HR[f]	1.00	0.99 (0.80–1.23)	1.36 (1.27–1.46)	1.49 (1.36–1.62)
Multivariable-adjusted HR[g]	1.00	0.97 (0.77–1.22)	1.42 (1.32–1.52)	1.51 (1.38–1.65)
Central area (n)	68,700 (83.6%)	2,055 (2.5%)	7,275 (8.9%)	4,147 (5.1%)
Sex- and age-adjusted HR	1.00	1.15 (1.06–1.25)	1.36 (1.31–1.41)	1.41 (1.36–1.47)
Multivariable-adjusted HR	1.00	1.10 (1.01–1.21)	1.38 (1.32–1.43)	1.44 (1.38–1.51)
Coastal area (n)	18,231 (84.2%)	627 (2.9%)	1,684 (7.8%)	1,113 (5.1%)
Sex- and age-adjusted HR	1.00	1.19 (1.05–1.35)	1.23 (1.15–1.33)	1.39 (1.30–1.49)
Multivariable-adjusted HR	1.00	1.29 (1.12–1.49)	1.29 (1.19–1.39)	1.46 (1.35–1.57)
Evacuation areas (n)	9,991 (87.8%)	176 (1.6%)	913 (8.0%)	304 (2.7%)
Sex- and age-adjusted HR	1.00	1.04 (0.79–1.37)	1.42 (1.29–1.55)	1.45 (1.28–1.64)
Multivariable-adjusted HR	1.00	1.13 (0.83–1.52)	1.43 (1.30–1.58)	1.49 (1.31–1.70)

[a]At-risk drinkers in women was defined as drinking ≥22 g of ethanol per day.

One "go" is equivalent to 22 g of ethanol and approximately "2 drinks."

[b]Neither were those who were not at-risk drinking pre-disaster (2008–2010) and post-disaster (2011–2012) and used as reference.

[c]Pre–disaster was defined as those who were at-risk drinking pre-disaster, but not post-disaster.

[d]Post–disaster was defined as those who were at-risk drinking post-disaster, but not pre-disaster.

[e]Both was defined as those who were at-risk drinking pre- and post-disaster.

[f]Sex- and age-adjusted HR was adjusted for age and sex.

[g]Multivariable-adjusted HR was adjusted for age and sex, body mass index, current smoking, regular exercise, sleeping habits.

eFigure 1. Age- and sex-adjusted proportion of heavy drinkers (≥66 g of ethanol) in men by geographic area from 2008 to 2017

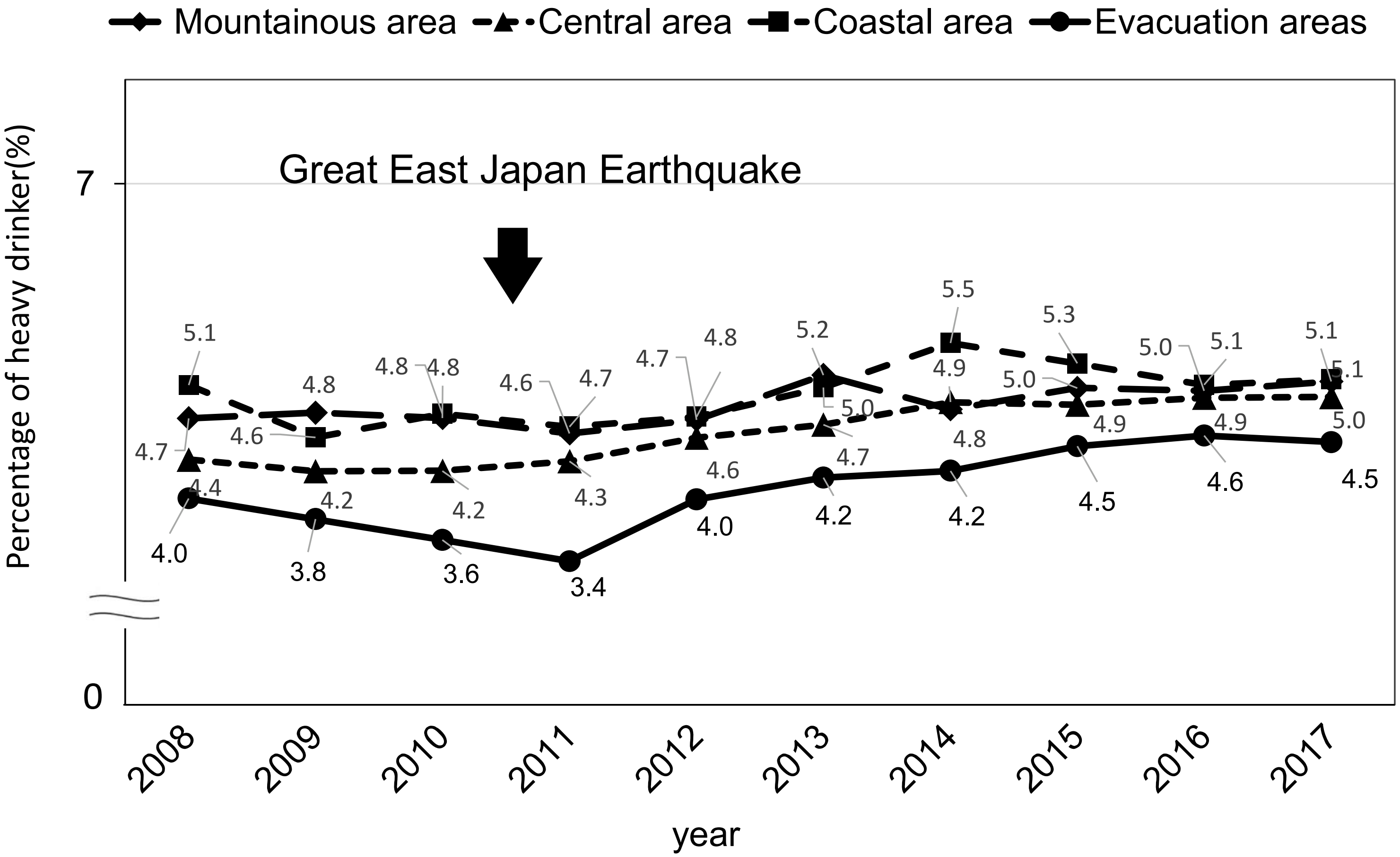

Age- and sex-adjusted proportion of heavy drinkers (≥66 g of ethanol) in Fukushima Prefecture by geographic area in men during 2008–2017.

Three “go” is equivalent to 66 g of ethanol and approximately “6 drinks.” Heavy drinkers in men was defined as drinking ≥66 g of alcohol per day, which is approximately corresponding to binge drinking or heavy episodic drinking.

eFigure 2. Age- and sex-adjusted proportion of at-risk drinkers (≥22 g of ethanol) in women by geographic area from 2008 to 2017

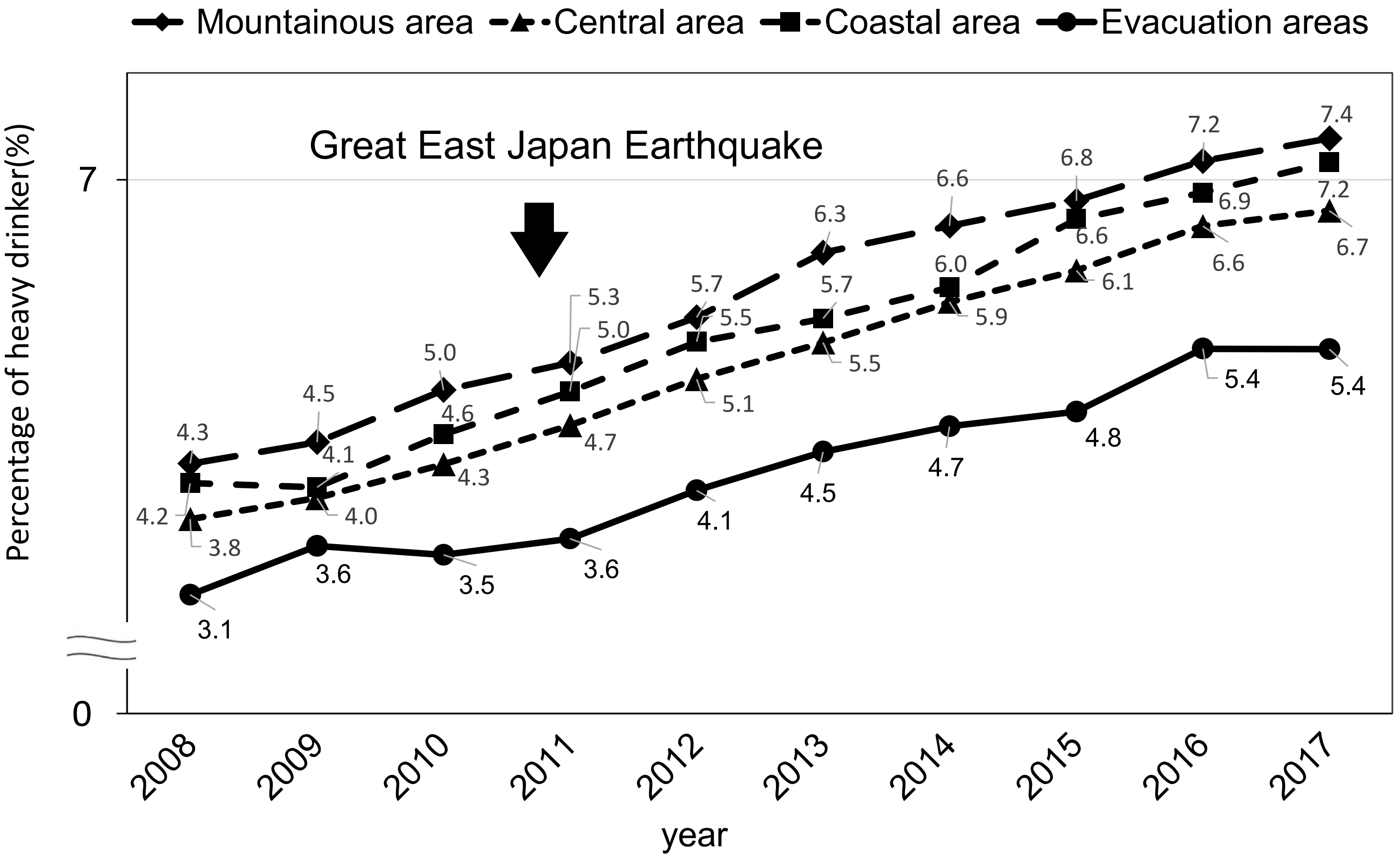

Age- and sex-adjusted proportion of at-risk drinkers (≥22 g of ethanol) in Fukushima Prefecture by geographic area in women during 2008–2017.

One “go” is equivalent to 22 g of ethanol and approximately “2 drinks.”

At-risk drinkers in women was defined as drinking ≥22 g of alcohol per day.
